# An Editorial on the Special Issue “Where Are We Now and Where Is Cell Therapy Headed?”

**DOI:** 10.3390/pharmaceutics17070894

**Published:** 2025-07-09

**Authors:** Andrea Papait, Paola Chiodelli

**Affiliations:** 1 Department of Life Sciences and Public Health, Università Cattolica del Sacro Cuore, 00168 Rome, Italy; 2 Fondazione Policlinico Universitario A. Gemelli IRCCS, 00168 Rome, Italy

Cell-based therapies have swiftly transitioned from experimental modalities to core components of modern translational medicine. Initially envisioned as a regenerative tool relying on engraftment and tissue integration, cell therapy has undergone a conceptual shift, and it is becoming increasingly evident that its therapeutic impact is rooted not in cellular persistence, but in the transient orchestration of complex biological responses.

A paradigmatic case is the evolution of mesenchymal stromal cells (MSCs), which have historically been employed for tissue regeneration but are now recognized for their immunomodulatory and paracrine effects [10.1002/sctm.17-0051]. The growing understanding of MSCs as secretory platforms has accelerated interest in cell-free therapeutic strategies, including extracellular vesicles (EVs) and complex secretomes [1,2,3]. These acellular approaches may mitigate issues related to immune rejection and engraftment failure while offering greater control and standardization.

Nonetheless, certain cell-based interventions remain clinically indispensable. Allogeneic hematopoietic cell transplantation (HCT), for example, is still a foundational therapy for hematological malignancies such as leukemia, lymphoma, and multiple myeloma [4,5]. At the same time, challenges such as graft versus host disease (GvHD) and donor availability have prompted the development of precision genome-editing approaches—most notably, CRISPR-Cas9—to reduce immunogenicity and correct autologous mutations [6].

In oncology, the success of engineered immune effectors—particularly chimeric antigen receptor (CAR) T-cells—has redefined expectations. Initially approved for specific forms of relapsed or refractory B cell malignancies, CAR T-cell therapies represent a new class of “living drugs” capable of sustained, antigen-driven immune activity [7,8]. Their efficacy has spurred efforts to extend the platform to solid tumors and integrate it with other immune-modulatory strategies.

Despite these advances, critical bottlenecks persist. Complex manufacturing pipelines, variability in product quality, nonuniform global regulatory frameworks, and cost-related access disparities all impede clinical scalability [9,10]. The articles in this Special Issue “Where Are We Now and Where Is Cell Therapy Headed?” reflect a translational field that confronts these challenges directly. Collectively, they map a landscape in which cellular and acellular approaches are no longer mutually exclusive but increasingly complementary.

Several contributions focus on the shift from autologous to allogeneic and off-the-shelf strategies. Jeannerat and colleagues demonstrate that FE002-derived progenitor cells retain chondrogenic potential and survive hypoxic and inflammatory stress in intervertebral disc models (Contribution 1). Extending their approach, they develop bioengineered neoligament constructs with FE002-derived tenocytes, achieving rapid fabrication and effective integration (Contribution 2). Philippe and colleagues compare autologous and allogeneic grafts for knee cartilage repair and find that allogeneic products reduce patient variability and enhance scalability without compromising clinical outcomes (Contribution 3). In their paper on burn care, Chen and colleagues describe a hybrid dermo-epidermal graft incorporating both autologous and banked fibroblasts, offering a more agile production timeline with sustained efficacy (Contribution 4).

In parallel, the therapeutic potential of secreted factors is gaining clinical traction. Wu and colleagues provide a comprehensive analysis of ESCs, iPSCs, MSCs, and retinal progenitors for treating degenerative retinal conditions, outlining both opportunities and translational barriers (Contribution 5). Within the cancer immunotherapy space, Li and colleagues. explore cellular strategies to modulate the tumor microenvironment (TME), including the reprogramming of tumor-associated macrophages (Contribution 6) and the enhancement of checkpoint inhibitor responses in non-small cell lung cancer (Contribution 7). Meanwhile, Chiodelli and colleagues demonstrate that conditioned medium from human amniotic MSCs suppresses ovarian cancer proliferation and migration, exhibiting enhanced efficacy in combination with paclitaxel (Contribution 8).

Together, these contributions illustrate a rapidly diversifying field. On the one hand, the development of standardized, bankable cell products reflects a drive toward consistency and broader accessibility. On the other hand, cell-derived EVs and secretomes are emerging as potent therapeutic agents, capable of modulating immune responses, promoting tissue repair, and engaging in cross-tissue signaling.

Regardless, considerable challenges lie ahead. Product heterogeneity remains a critical concern for both cellular and extracellular therapies [11,12], and the lack of universally accepted potency assays and markers of activity further complicates development [13]. Issues of manufacturing scalability [9], cost containment [14,15,16], and international regulatory alignment [10,17,18] continue to shape—and constrain—the pathway from bench to bedside.

Nevertheless, the field is clearly beginning to gravitate toward therapies that extend beyond the cells themselves. The next generation of interventions is likely to harness not the cell as a unit, but the molecular and vesicular cargo it deploys. This reframing positions cell therapy as a modular, signal-driven platform, which is defined less by cellular identity and more by the biological programs it initiates within the host.

## Abbreviations

The following abbreviations are used in this manuscript:

MSC	Mesenchymal Stromal Cells
HCT	Hematopoietic cell transplantation
ESC	Embryonic Stem Cells
GvHD	Graft Versus Host Disease
iPSC	Induced Pluripotent Stem Cells
TME	Tumor Microenvironment
EV	Extracellular Vesicle
CAR	Chimeric Antigen Receptor
